# Pre-Trial EEG-Based Single-Trial Motor Performance Prediction to Enhance Neuroergonomics for a Hand Force Task

**DOI:** 10.3389/fnhum.2016.00170

**Published:** 2016-04-25

**Authors:** Andreas Meinel, Sebastián Castaño-Candamil, Janine Reis, Michael Tangermann

**Affiliations:** ^1^Brain State Decoding Lab, Cluster of Excellence BrainLinks-BrainTools, Department of Computer Science, Albert-Ludwigs-UniversityFreiburg, Germany; ^2^Department of Neurology, Albert-Ludwigs-UniversityFreiburg, Germany

**Keywords:** single-trial performance prediction, trial-by-trial variability, isometric force modulation, hand motor rehabilitation, visuomotor integration, EEG, oscillatory subspace, spatial filtering

## Abstract

We propose a framework for building electrophysiological predictors of single-trial motor performance variations, exemplified for SVIPT, a sequential isometric force control task suitable for hand motor rehabilitation after stroke. Electroencephalogram (EEG) data of 20 subjects with mean age of 53 years was recorded prior to and during 400 trials of SVIPT. They were executed within a single session with the non-dominant left hand, while receiving continuous visual feedback of the produced force trajectories. The behavioral data showed strong trial-by-trial performance variations for five clinically relevant metrics, which accounted for reaction time as well as for the smoothness and precision of the produced force trajectory. 18 out of 20 tested subjects remained after preprocessing and entered offline analysis. Source Power Comodulation (SPoC) was applied on EEG data of a short time interval prior to the start of each SVIPT trial. For 11 subjects, SPoC revealed robust oscillatory EEG subspace components, whose bandpower activity are predictive for the performance of the upcoming trial. Since SPoC may overfit to non-informative subspaces, we propose to apply three selection criteria accounting for the meaningfulness of the features. Across all subjects, the obtained components were spread along the frequency spectrum and showed a variety of spatial activity patterns. Those containing the highest level of predictive information resided in and close to the alpha band. Their spatial patterns resemble topologies reported for visual attention processes as well as those of imagined or executed hand motor tasks. In summary, we identified subject-specific single predictors that explain up to 36% of the performance fluctuations and may serve for enhancing neuroergonomics of motor rehabilitation scenarios.

## 1. Introduction

Motor training is utilized in rehabilitation scenarios to accelerate the re-gain of lost motor function after brain injury. State-of-the-art rehabilitation concepts are based on repetitive training tasks with the aim to reach a functional gain (Dobkin, 2004; Timmermans et al., 2009; Langhorne et al., 2011). Most prominent training paradigms comprise mirror training (French et al., 2007), constraint-induced movement therapy (Wolf et al., 2002), simultaneous bilateral training (Coupar et al., 2010), BCI-supported training (Ang and Guan, 2013) and robot-assisted techniques (Kwakkel et al., 2008). Recent rehabilitation approaches include the training of novel, unfamiliar motor skills instead of training well-known habitual motor tasks, attempting to optimize functional cortical reorganization.

Repetitive paradigms allow for the assessment of motor performance on a very fine-granular time scale. The performance of each single trial can be monitored by metrics such as the length, speed or smoothness of the produced movement trajectory. The distributions and temporal characteristics of trial-wise motor performance variations have been studied by different groups (Abe and Sternad, 2013; van Beers et al., 2013; Wu et al., 2014; Hadjiosif and Smith, 2015). While practicing a motor task over several sessions enables a user for skill acquisition (Lage et al., 2015), trial-by-trial variability of motor performance is a prominent feature which does not fully vanish with training (Cohen and Sternad, 2009; Osu et al., 2015). The underlying neural mechanisms of motor performance fluctuations on short time scales is subject of controversial discussion in literature and is not fully resolved yet (Faisal et al., 2008; Hadjiosif and Smith, 2015; Osu et al., 2015).

In the present work, we aim toward closing this gap. Therefore, trial-wise performance fluctuations of a sequential visuo-motor task (SVIPT; Camus et al., 2009; Reis et al., 2009; Fritsch et al., 2010 are investigated while registering a user's brain activity by EEG. In SVIPT trials, the quality of a movement changes within seconds and from repetition to repetition.

Our *hypothesis* is that subject-specific pre-trial brain signals can partially explain and temporally predict the trial-by-trial fluctuations of the upcoming motor performance. Given that such informative neural markers exist, then the SVIPT paradigm could be altered in order to meet the cognitive ergonomic requirements of each single user. Practically, the starting time point of the upcoming trial can be determined based on the information contained in this pre-trial neural marker. Ideally, such a neuroergonomic closed-loop gating strategy could provide control over the level of difficulty. This should allow to causally influence user performance and ultimately support SVIPT motor learning on the long run.

Paradigms which include *brain-state-dependent experimenting* (see Jensen et al., 2011; Horschig et al., 2014c) require that an informative neural marker can be extracted robustly from brain signals. Given the high dimensionality and noisy characteristic of most types of brain signals, the extraction and decoding of such individual neural markers is a challenging task.

Screening literature on relevant neural markers of visual and motor performance, it is important to make a distinction between the use of single-trial decoding in contrast to the extraction of statistical differences, which may even be reported as group averages. Neural features which correlate with the task performance on the grand average (GA) of a set of subjects have limited usefulness for closed-loop experimenting with a given individual. As inter-subject differences get lost during the averaging, GA features may have low predictive power when tested with data of a novel subject. Research in the field of brain-computer interfaces (BCI) has pushed forward methods for single-trial decoding of individual brain activity (mostly EEG signals) (Millán et al., 2010; Makeig et al., 2012). Results from this field affirm that brain signals and informative features vary strongly between individuals (Müller et al., 2008). To obtain optimal decoding results, BCI data processing pipelines thrive to identify subject-specific informative features. Technically, these are gained either from a calibration recording prior to the online use of the BCI (Blankertz et al., 2007), or by transfer learning methods (Kindermans et al., 2014) which exploit features from pre-trained machine learning models of earlier sessions or previous users. Furthermore, attention needs to be paid to temporal dependencies: brain features may correlate with previous behavior, with simultaneous behavior or may even be predictive for future behavior. Only the latter brain features can serve as a tool for brain-state-dependent experimenting.

Statistical correlates of visual perception performance are reported by several groups. For stimuli near the perception threshold, the pre-stimulus occipital alpha bandpower correlates with the detection performance (van Dijk et al., 2008), even on a single-trial basis using predictive features (Hanslmayr et al., 2007). In addition to bandpower, the pre-stimulus alpha phase was reported to correlate with the detection performance (Busch et al., 2009). Single-trial decoding methods were not applied in those auditory studies, but the reported correlates precede the perception, which may open the possibility for closed-loop experimenting. Based on the findings of Hanslmayr et al. (2007) and van Dijk et al. (2008), there are two examples that set up an online experiment based on occipital alpha bandpower features. Tonin et al. (2013) using EEG data and Horschig et al. (2014b), who employed MEG signals, both decoded covert visual attention in a closed-loop experiment by utilizing single-trial feedback on the detected attention shift. However, both groups did not fully close the loop e.g., by manipulating the perception performance, which may have been possible by selecting suitable brain states for stimulation. Gonzalez Andino et al. (2005) studied a cued reaction time task and identified that gamma band oscillatory activity observed in fronto-parietal regions prior to the stimulus onset correlates with reaction time. Similarly, Hoogenboom et al. (2010) stated that the strength of visually induced gamma band activity is predictive for the detection of stimulus motion. Somatosensory stimuli of low-intensity, but above threshold were delivered and combined with a distracting masker stimulus by Schubert et al. (2009). Investigating perceived vs. missed stimuli in an offline analysis, pre-stimulus beta bandpower over the left frontal cortex was found predictive for the perception performance on the grand average, as well as mu and beta bandpower over the pericentral sensorimotor areas.

In the motor domain, several groups have successfully decoded hand kinematics, using the center-out task as the dominating experimental approach. In their own work, Jerbi et al. (2011) provide a review over the decoding of hand movement parameters such as direction, position and velocity based on brain signals. ECoG signals were used by Pistohl et al. (2008) to decode two-dimensional hand movement trajectories using an autoregressive filtering approach. Considering non-invasive techniques, Waldert et al. (2008) have decoded (but not temporally predicted) the hand movement direction based on MEG and EEG. Neural correlates which encode the velocity of a movement have been investigated by Bradberry et al. (2009). The decoding of produced grip force based on a phase feature extracted from the beta range has been reported on data of three subjects by Logar et al. (2008). Zaepffel et al. (2013) reported an increased centro-parietal beta power during the planning period of grasping movements, but it was not investigated, if decoding may work on the basis of single trials. Focusing on single-trial methods, Lew et al. (2014) used slow cortical potentials of the EEG from fronto-parietal areas to predict self-paced movement directions a few hundred milliseconds prior to movement onset. Similarly, Hammon et al. (2008) inspected predictive EEG features for planning target directions using a cue-based paradigm.

In the field of BCI research, Maeder et al. (2012) studied a motor imagery paradigm. The single-trial decoding performance of left vs. right hand movement imagery tasks could be correlated to the level of pre-trial alpha bandpower over the sensorimotor cortices. Despite used offline, this neural marker would allow for a predictive intervention in a closed loop. In their statistical analysis, Yang et al. (2014) identified frontal alpha and beta bandpower features which correlate with performance metrics of a reaching task. Proceeding to single-trial methods, Meyer et al. (2014) reported on data of six subjects, who performed a hand positioning task. Their offline analysis revealed that the normalized time-to-target could be predicted based on pre-cue alpha-band activity of the EEG.

The state-of-the-art can be summed up as follows: In the perception domain, several studies have established single-trial performance prediction, partially even in closed-loop applications. The situation is different for the motor domain since only very few studies have investigated subject-specific motor performance prediction in single-trial upon a sufficiently large subject group. Closest to all of these requirements is the study by Meyer et al. (2014). Our research hypothesis builds exactly upon this point. In the context of a hand force task, we propose a generalized *workflow* which identifies subject-specific *predictive* oscillatory EEG features evaluated on a single trial basis.

First, by means of a simulated online analysis, an approach to extract robust and meaningful EEG components is developed. We evaluate, if the information contained in selected components is able to partially explain the trial-by-trial variation of SVIPT performance in a predictive fashion, i.e., the pre-trial component is required to predict the outcome of the upcoming trial. Second, the characteristics of the best performance predictors are investigated by a group-level analysis.

## 2. Materials and methods

### 2.1. Hand motor paradigm

**Figure 1:**
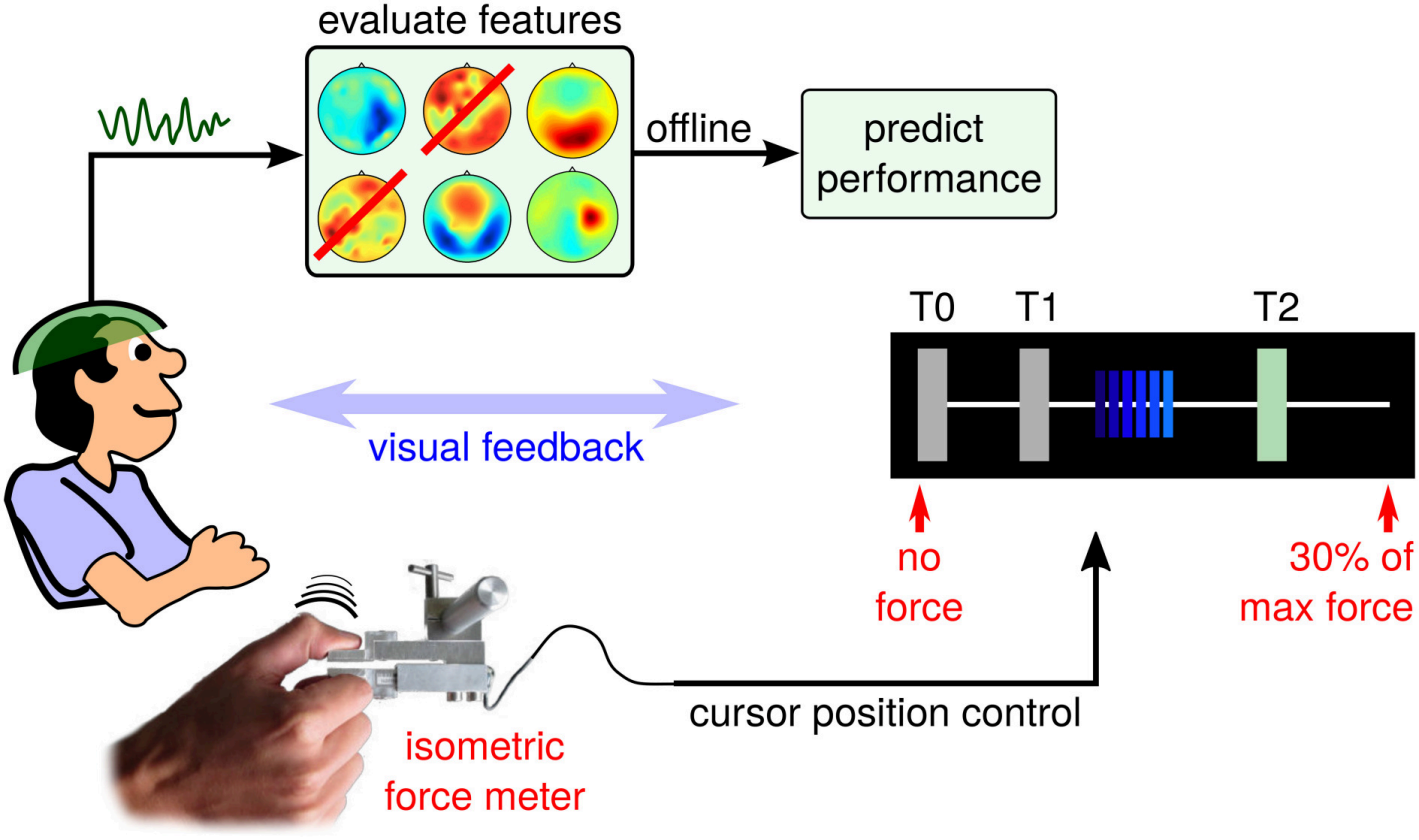
**Schematic setup of the EEG-tracked SVIPT**. The subject applies force to a sensor using a pinch grasp. Force is transduced into positions of a horizontally moving cursor. EEG activity is recorded before, during and after repeated trials.

In the context of hand motor skill learning, Reis et al. (2009) investigated the *Sequential Visual Isometric Pinch Task* (SVIPT), which demands an isometric force control of thumb and index finger. Interestingly, training-induced improvement of the SVIPT generalizes well to other hand motor control tasks, even though pinch grasp activities are rarely displayed during natural behavior patterns. Compared to the original SVIPT setup, brain activity is recorded using electroencephalogram (EEG) during a training session for *post-hoc* offline analysis. The resulting SVIPT setup follows the proposal in Meinel et al. (2015) and is sketched in Figure 1.

Each SVIPT trial consists of three phases: a light blue (inactive) cursor appears on the leftmost edge of the T0 field (corresponding to zero force), while the user is touching the sensor only slightly with his non-dominant left hand. The appearance of the cursor indicates the start of the *get-ready* phase, which corresponds to a waiting period with enhanced attention level. Its duration is varied randomly between 2 and 3 s. The transduction of force into cursor movements is deactivated during the get-ready phase. Fixating the cursor, the user will observe a distinct color change of the cursor from light to dark blue. This *go-cue* indicates the beginning of the *running* phase, in which the cursor position can be controlled by applying force to the sensor. As force is transduced into horizontal cursor position, increasing force will move the dark blue cursor to the rightmost position, which is pre-calibrated at session start to represent 30% of the user's maximum force. The user has been instructed to navigate the cursor as quickly and accurately as possible, in order to visit a sequence of target fields (T0, T1, and T2). Overshoots of the cursor are to be avoided. The current target field is visually indicated to the subject by a green shading (see Figure 1). Reaching a target field, a dwell time of 200ms must be fulfilled in order to achieve a successful hit of this target field. Hit events are indicated visually by a switch of the target field (another field is shaded in green), or by the end of the trial. Trials were chosen randomly from two conditions, each with a specific required target field sequence (T1-T0-T2-T0 or T2-T0-T1-T0). A trial was finished by fulfilling the complete sequence – skipping a target was not allowed. Trial duration is presented visually as an immediate performance feedback during the *pause* phase between trials.

### 2.2. Subjects and ethics

Overall, 20 right-handed *normally aged* subjects (8 female, average age: 53 years, std: 6 years) were recruited. The subject group resembles the target group of first-stroke patients with respect to age and gender (Ovbiagele and Nguyen-Huynh, 2011). The term *normally aged* was chosen to indicate our selection criteria: the participants did not have any known neurological or psychological history and were probably healthy—even though we can not exclude the possibility that some participants had a history of unrecognized micro stroke events.

The offline study was approved by the Ethics Committee of the University Medical Center Freiburg. Following the principles of the Declaration of Helsinki, written informed consent was given by subjects prior to participation. In one session of about 3–4 h (including EEG setup and washing the hair), every participant controlled the cursor with their left hand for 20 blocks of 20 trials each.

### 2.3. SVIPT performance scores

SVIPT enables to capture single trial motor performance. Given a high order motor control, the force profile *F*(*t*) of a single-trial is characterized by a quick force ramp up upon the presentation of the go-cue and the avoidance of overshoots. The requested speed-accuracy trade-off can be translated into various performance scores of the SVIPT task. In Tangermann et al. (2015), the authors selected a set of scores, which describe the single-trial performance:

- **Reaction Time/RT**: A quick response upon the *go-cue* is a good start for a successful trial. The time interval between the *go-cue* at time *t*_*go*_ and the time point *t*_*T*0, *exit*_, which indicates the cursor leaving the starting field T0, is defined as reaction time.
- **Duration/DUR**: Comparable to RT, a short time duration from the *go-cue* at time *t*_*go*_ until the hit of the first target field at time point *t*_*hit*_ characterizes a successful trial.
- **Cursor Path Length/CPL**: The total path length the cursor is moved from the *go-cue* to the hit of the first target field is described by the integral over the first temporal derivative of the force profile *F*(*t*):
  $$CPL\equiv \int_{t_{go}}^{t_{hit}}|\dot{F}(t)|dt^{\prime }$$
- **Integrated Squared Jerk/ISJ**: The level of fine-granular motor control is reflected in variations of the trajectory smoothness. Therefore, jerk—defined as the third derivative of the force profile—is expressed by the ISJ metric, which is defined as:
  $$ISJ\equiv \int_{t_{go}}^{t_{hit}}|\frac{d^{3}F(t)}{dt^{3}}{|}^{2}dt^{\prime }$$
- **Normalized Jerk/NJ**: A unit-free variant of ISJ captures smoothness variations. It is given by the *normalized* jerk:
  $$NJ\equiv \sqrt{\frac{ISJ\;\cdot \;DUR^{5}}{2\;\cdot \;CPL^{2}}}$$

Since there are two conditions of target field sequences, a standardization of the performance scores (except for RT) is the prerequisite for pooling trials of both conditions. Therefore, the extracted metrics of each condition were standardized (zero mean and standard deviation one) prior to pooling. Except RT, the metrics are defined with respect to some end point (e.g., *t*_*hit*_). Choosing this boundary represents a trade-off between (a) harvesting a metric which is temporally close and thus related to the get-ready interval (the interval before the *go-cue*), and (b) including thorough information about the force trajectory of the current trial. To balance the two conflicting goals, we chose the hit of the first target field.

### 2.4. Data acquisition and preprocessing

During a single session, subjects were placed in a chair at 80 cm distance from a 24-inch flat screen. EEG signals from 63 passive Ag/AgCl electrodes (EasyCap) were recorded, which were placed according to the extended 10–20 system. Impedances were kept below 20 *kΩ*. All channels were referenced against the nose. The signals were registered by multichannel EEG amplifiers (BrainAmp DC, Brain Products) at a sampling rate of 1 kHz. An analog lowpass filter of 250 Hz was applied before digitization. The signal of the force sensor was recorded by an additional amplifier system (BrainAmp ExG, Brain Products).

For outlier identification, the offline preprocessing consisted of high-pass filtering the raw EEG signals at 0.2 Hz, low-pass filtering at 48 Hz and sub-sampling to 500 Hz sample frequency. Therefore, linear butterworth filters of 5th order were applied. For each trial and all 63 channels, an epoch of 2000 ms duration prior to the *go-cue* was extracted. In order to identify outlier epochs, three rejection methods were applied. First, EEG epochs violating a min-max threshold of 60μ*V* on frontal channels were excluded from further analysis. Second, a variance threshold on single epochs and channels was applied to remove high-frequent muscular artifacts. Therefore, the variance of single epochs needs to be within *P*_*up*_ = 90th percentile and is not allowed to exceed 2·(*P*_*up*_ − *P*_*low*_) with *P*_*low*_ = 10th percentile. Third, epochs belonging to extreme trials, represented by outliers of the motor performance metric, were removed. For this purpose, the following min-max thresholds were defined based on earlier pilot recordings (Meinel et al., 2015). The thresholds were [150, 900] ms for RT, [−1.5, 1.5] for ISJ, [−0.6, 0.6] for CPL, [−1.5, 2] for DUR, and [0, 1300] for NJ. They were applied prior to further data analysis. The total number of trials *N*_*e*_ entering the following offline analysis procedures varied across subjects and performance metrics. Only for 2 out of 20 subjects less than 150 out of the original 400 epochs were remaining after the EEG preprocessing. We discarded data of these subjects from the following analysis. The frequency filtering for our main analysis will subsequently be described in Section 2.6.

### 2.5. Single-trial performance prediction

In the following, the multivariate variable $\text{x}\left(t\right)\in {\mathbb{R}}^{N_{c}}$ characterizes the EEG signal recorded from *N*_*c*_ sensors. In addition, **s**(*t*) defines the time course of a neural source. The physics of volume conduction assumes a linear mapping of the source space to the sensor space. The forward (or generative) model thus reads :

(1) x(t)=A s(t)

where the matrix $\text{A}\in {\mathbb{R}}^{N_{c}\times M}$ describes the projection of the M sources to the EEG sensor space.

The main goal is to approximate the true neural source **s**(*t*) by $\hat{\text{s}}\left(t\right)$, whose power achieves the highest correlation with a predefined external variable *z*(*t*), called *target variable* from this point onwards. Several methods can be used to estimate such a source, among them Blind Source Separation (BSS) and source reconstruction techniques. BSS techniques rely solely on an unsupervised framework, which may be a suboptimal approach given the availability of labels in the form of the target variable *z*. Source reconstruction techniques may provide a high level of interpretability for the results directly in the source space, and may describe non-stationarities in the data and other complex dynamics (Castaño-Candamil et al., 2015b). However, there are three potential drawbacks of source reconstruction approaches (Grech et al., 2008): First, the estimation of $\hat{\text{s}}\left(t\right)$ usually creates a rather high computational burden. Second, the methods require a forward model **A** for each individual subject, which may not be available in most situations since it corresponds to the exact anatomical description of a subject's brain. Third, as source reconstruction problems are intrinsically ill-posed, the quality of an estimated source depends on additional assumptions, such as the density of sources or their location within the brain.

An alternative to both, BSS and source localization approaches is the family of the so-called supervised spatial-filtering methods. One widely known approach is the Common Spatial Patterns algorithm (CSP; Ramoser et al., 2000), which searches for spatial filters that enhance the contrast between two classes. Consequently, it is well suited for supervised classification problems. A more recent approach, the Source Power Comodulation algorithm (SPoC; Dähne et al., 2014) is adequate for regression problems. As the five SVIPT metrics are continuous variables, we preferred to include the SPoC algorithm into our data analysis framework.

SPoC learns an optimal spatial filter $w_{opt}\in {\mathbb{R}}^{N_{c}\times 1}$ that allows to estimate the source as $\hat{\text{s}}\left(t\right)$ by projecting **x**(*t*) into a subspace, which maximizes the correlation between the band power of $\hat{\text{s}}\left(t\right)$ and the target variable *z(t)*:

(2) $$\hat{s}(t)=w_{opt}{}^{⊤}x(t)$$

Without loss of generality, the objective function for SPoC may be defined in terms of the epoched data **x**(*e*), where *e* refers to the *e*-th epoch. Assuming that the EEG sensor signal has been bandpass-filtered to a narrow frequency band and that the norm of the spatial filter is constrained, e.g., $\mathrm{Var}\left[{\text{w}}^{⊤}\text{x}\left(t\right)\right]\overset{!}{=}\;1$, the optimization problem can be solved by maximizing the covariance:

(3) $$\begin{array}{l} w_{opt}=\underset{w}{\mathrm{argmax}}\{\mathrm{Cov}[\Phi_{x}(e),\;z(e)]\}\;\forall \;e\\ \text{s}.\text{t}.\mathrm{Var}[w^{⊤}x(t)]\overset{!}{=}1 \end{array}$$

where Φ_*x*_(*e*) = Var[***ŝ***](*e*). This formulation of the algorithm – called SPoC_λ_ – can be transformed into a generalized eigenvalue problem and thus delivers a set of *N*_*c*_ spatial filters $\text{W}\in {\mathbb{R}}^{N_{c}\times N_{c}}$. In this paper, the SPoC_λ_ algorithm is utilized which subsequently will be abbreviated by the term SPoC.

Applying a SPoC filter **w_tr_** learned from training data **x_tr_**(*t*), the method allows to estimate the target variable *z*_*est*_ on novel, unseen test data **x_te_**(*t*) on a single-trial basis by calculating the bandpower of the narrowband subspace signal:

(4) $$z_{est}=\mathrm{Var}[w_{tr}{}^{⊤}x_{te}(t)](e)$$

Using this relation, we will focus on the prediction of single-trial SVIPT performance using EEG activity within the *get-ready* phase of the trial.

As Haufe et al. (2014) have been pointing out, there is an existing forward model of the form of Equation (1) for every backward model as in Equation (2). Thus, the corresponding spatial activation patterns can be obtained from the spatial filters **W** via the covariance matrix **C_x_** of the data **x**(*t*) via:

(5) $$A=C_{x}W$$

Note, that any subspace components resulting from the SPoC analysis depend mainly on four hyperparameters. In the temporal domain, two of them define the epoching interval [*t*_0_, *t*_0_ + Δ*t*] where *t*_0_ is the starting time relative to the *go-cue* and a duration Δ*t*. In the frequency domain, the lower frequency *f*_0_ and the band width Δ*f* are the hyperparameters describing the band [*f*_0_, *f*_0_ + Δ*f*] in which **x**(*t*) is bandpass-filtered.

Even though simple regression of bandpower features on the channel level does not fulfill the requirements of the assumed forward model, we added this simple method for comparison with SPoC. Therefore, channel-wise bandpower features of the training and test set were calculated.

### 2.6. Selection criteria for informative oscillatory components

Performing a grid search across subjects and SPoC parameters, we restrict the evaluation to a fixed predictive time interval given by *t*_0_ = −800 ms prior to the *go-cue* and a window size of Δ*t* = 750 ms.

**Figure 2:**
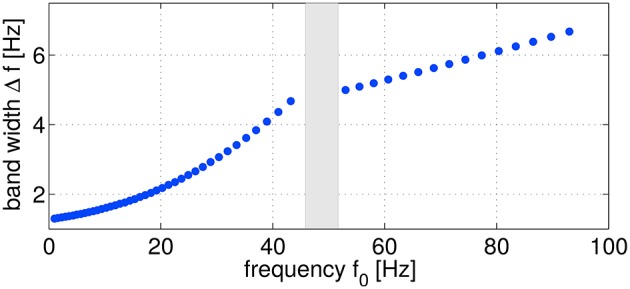
**Frequency parameter configurations characterized by the frequency *f*_0_ and the corresponding band width Δ*f***. In total, 55 configurations were used for computing SPoC filters. The omitted points (gray area) correspond to the power line frequency range.

As sketched in Figure 2, logarithmically increasing and overlapping frequency bands ranging from ≈ 1–100 Hz (55 configurations in total) were evaluated from the original non-filtered signals. For bandpass filtering, linear butterworth filters of 5th order were utilized. As a trial-wise target variable *z*, the five different performance metrics introduced in Section 2.3 were considered. Evaluating SPoC across the complete study group of 18 subjects, using five different motor performance metrics, sweeping through 55 discrete frequency bands and selecting the highest-ranked components (see details below) per configuration, results in more than 12,000 oscillatory components. In this section, we will describe an offline selection strategy in order to identify a subset of the most robust and informative oscillatory components which qualify to predict single-trial motor performance.

Upon each parameter configuration, a K = 5-fold chronological cross-validation procedure was employed upon the calculation of SPoC (Lemm et al., 2011). Only trials were considered, which survived the data preprocessing (see Section 2.4). From these, *N*_*e*_ EEG pre-trial epochs and their corresponding target variable values *z* were extracted in chronological order and split into 5 equally-sized folds. Thus, 4-folds served as training data while the remaining one was used for validating the SPoC filter as described in Equation (5). Since each fold served as test fold once, the estimated target variable *z*_*est, j*_ of fold *j* can be concatenated for all *N*_*e*_ epochs, resulting in *z*_*est*_ = [*z*_*est, j*_]_*j* ∈ [1, *K*]_. According to Equation (5), on each fold *j* the corresponding test pattern is given as **a_j_** = **C_te, j_w_tr_** utilizing the covariance matrix **C_te_** of the test data **x_te_**(*t*).

The same cross-validation scheme was applied upon the linear regression model. The whole parameter space of 3600 configurations was screened. Note, that this number is smaller than the number of components delivered by SPoC analysis, since the latter may deliver more than one component per parameter configuration. The regression, which delivers a single component per configuration only, was trained on the training data and finally applied on test data such that an estimate *z*_*est*_ was gained on all *N*(*e*) trials which had survived the data preprocessing step.

For a given parameter set, SPoC_λ_ returns a set of *N*_*c*_ filters. As described in Tangermann et al. (2015), it is sufficient to take only the first-ranked components into consideration^1^. For this purpose, we applied a rank-based criterion. First, removed the linear trend from the ordered set of *N*_*c*_ eigenvalues. A threshold of 1.5·σ(*r*) relative to the standard deviation σ of the resulting *N*_*c*_ residuals *r* was defined. We restricted the investigation to positive eigenvalues.

Given a single component **w**, the following set of scores enable to characterize its predictive strength and stability:

- **Correlation characteristics:** As a measure to verify the quality of the predictive strength of a SPoC configuration, the overall correlation of the *N*_*e*_-many measured performance labels *z*_*true*_ with the corresponding predictions *z*_*est*_ can be considered:
  (6) $$R_{all}=\mathrm{Corr}[z_{true},z_{est}]$$
  Similarly, the predictive strength in terms of single-trial performance can also be verified by checking the mean of the fold-wise correlations *R*_*j*_ = Corr[*z*_*true, j*_, *z*_*est, j*_], which rewards temporally stable components:
  (7) $$R_{folds}=\frac{1}{K}\sum_{j=1}^{K}\mathrm{Corr}[z_{true,j},z_{est,j}]$$
  The correlation based metrics *R*_*all*_ and *R*_*folds*_ come closest to the original optimization objective of the SPoC algorithm. If the trained spatial filters model trial-to-trial fluctuations well, *R*_*all*_ and *R*_*folds*_ will report a large value, but only *R*_*folds*_ allows to discriminate between single-trial predictors and session-trend models. Furthermore, a stable component requires that the correlation of each fold *j* shares the same sign with *R*_*all*_. Thus, it is reasonable to require a high homogeneity *H*_*folds*_:
  (8) $$H_{folds}=\sum_{j=1}^{K}\Theta (\mathrm{sign}(R_{all})\;\cdot \;\mathrm{sign}(R_{j})),$$
  with Θ(*x*) = 1 for *x* ≥ 0 and Θ(*x*) = 0 for *x* < 0 representing the unit-step function.
- **Separability of estimated performance:** Simulating the trial-wise online application, the continuous prediction is transferred into a two-class problem. Therefore, we split the *N*_*e*_ prediction values *z*_*est*_ into two distributions (*z*_*est, h*_ and *z*_*est, l*_) based on the 50th percentiles of the true target variable distribution from *z*_*true*_, thus representing high and low performance. The separability of *z*_*est, h*_ and *z*_*est, l*_ can be quantified by a statistical test. Here, the area under the receiver-operating-characteristic curve (Fawcett, 2006) is reported. It is denoted as z-AUC and has a chance level of 0.5.
- **Stressing the stability:** SPoC is a supervised method, which uses label information to guide the spatial filter calculation. Thus, the robustness of a resulting component can be stressed by introducing label noise. The concept of a step-wise reduction of the SNR of the labels has been introduced by Castaño-Candamil et al. (2015a). Here, SNR levels were varied from −20 dB to 10 dB by adding white noise. Applying SPoC, we estimated the target variable *z*_*est*_ for all *N*_*e*_ epochs using 5-fold cross-validation. At each SNR level, three sets of noisy labels *z* were calculated. For each SNR level, the separability of the resulting *z*_*est*_ distribution is verified by the z-AUC value. Regarding the z-AUC values as a function of the SNR, the area under this curve—referred to as AAUC_SNR_—describes the stability of the component.

To finally identify and select robust and predictive components, we propose to apply three out of these five criteria in parallel. As a prerequisite, the data set needs to consist of at least *N*_*e*_ = 150 trials in order to ensure the convergence of the SPoC algorithm (see Dähne et al., 2014; Castaño-Candamil et al., 2015a):

1. The separability of the predicted performance *z*_*est*_ can be verified by the resulting z-AUC value. A corresponding threshold z-AUC_*min*_ = 0.59 was determined according to the 85th percentile across all configurations.
2. The stability of the component is assessed by the AAUC_SNR_. Here, a threshold AAUC_*min*_ = 0.18 was determined from the 85th percentile.
3. As an additional stability criterion, we require all fold-wise correlations *R*_*j*_ to share equal sign as *R*_*all*_ such that H_*folds, min*_ = 5.

## 3. Results

### 3.1. SVIPT performance metrics

**Figure 3:**
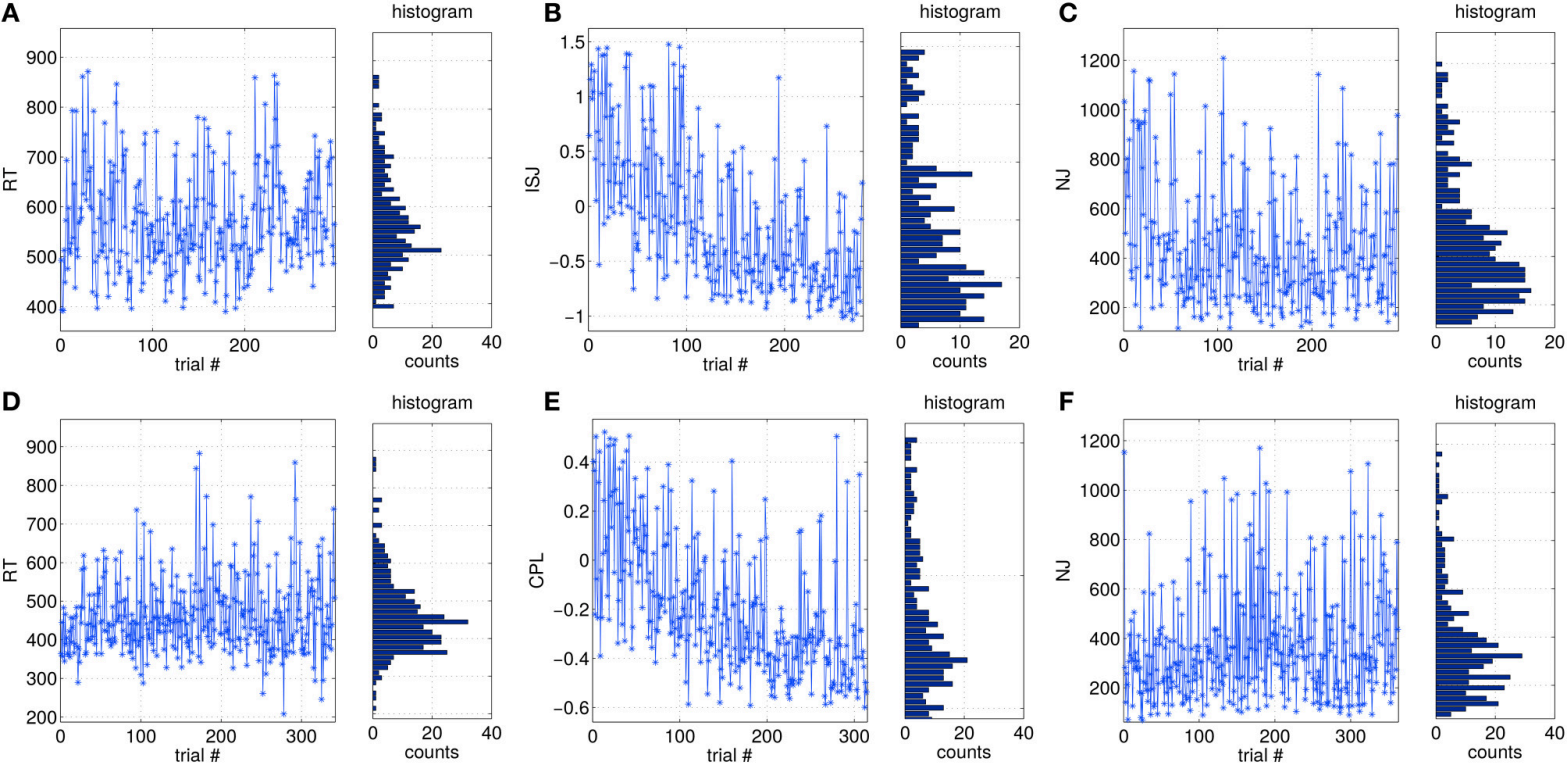
**Examples of trial-wise variations of different performance metrics over a full session, and their distributions**. **(A–C)** are taken from data of subject S9, while **(D–F)** are from S13.

Single-trial based SVIPT performance can be assessed by different metrics, as described in Section 2.3. In Figure 3 examples of the trial-to-trial fluctuations of different metrics are visualized for two subjects. The visualization covers the full sessions, but omits trials removed during the preprocessing. Figures 3A,D show the metric reaction time (RT) for two subjects. It is not affected by a session trend. Its distribution is slight asymmetric, which is caused by a physiological limit for the minimal RT. The normalized jerk (NJ) in Figures 3C,F behaves in a similar manner. It is affected only slightly by a global trend, but shows a more skewed distribution compared to RT. In contrast, integrated squared jerk (ISJ) depicted in Figure 3B, and cursor path length (CPL) in Figure 3E both show a strong session trend, which can be explained by the user learning (data not shown here). A comparably strong session trend is present also in the duration metric DUR (data not shown).

The cross-correlations between all five metrics and the shape of their distributions were reported in Tangermann et al. (2015). Metrics ISJ, CPL and DUR showed strong correlations to each other, while RT as well as NJ both are rather independent from the four other metrics.

### 3.2. Contrasting SPoC with linear regression on sensor level

**Figure 4:**
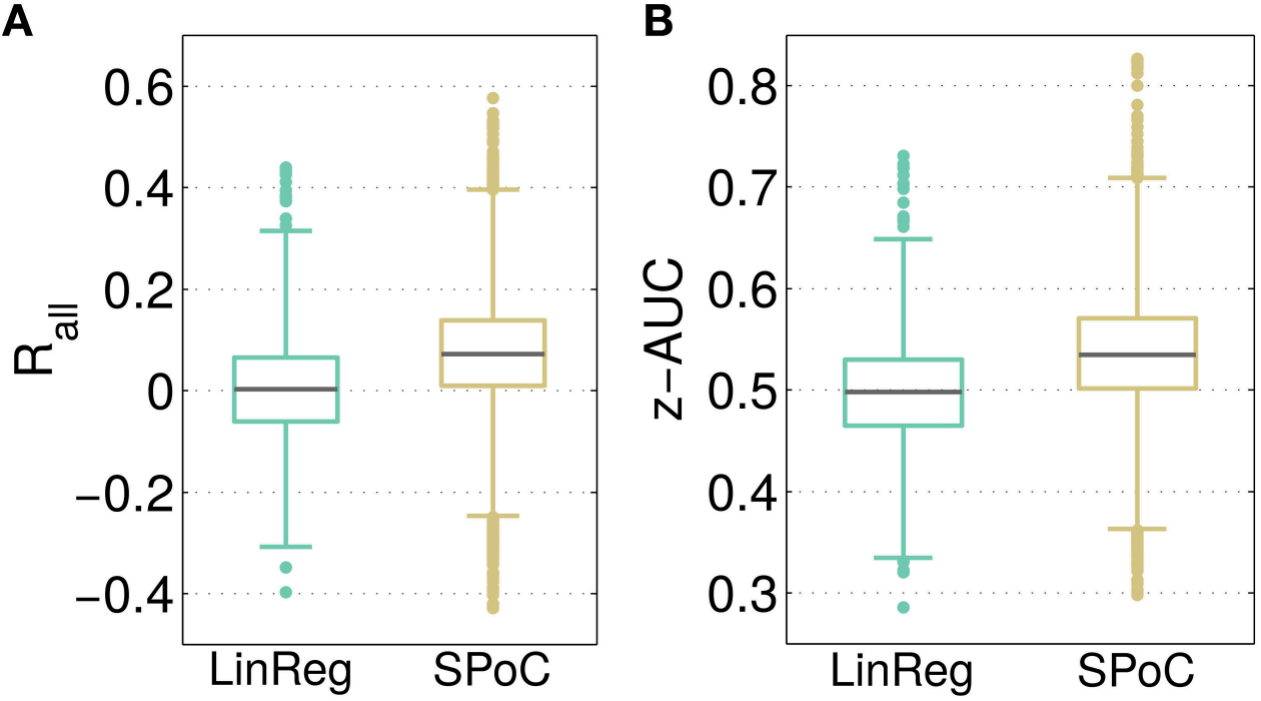
**Contrasting the predictive outcome of all 3600 tested parameter configurations for linear regression (LinReg) and over 12,000 configurations for SPoC**. In **(A)**, the overall correlation *R*_*all*_ between predicted and estimated target variable values is depicted. **(B)** Shows the performance separability z-AUC. Gray lines indicate the median, boxes enclose the 25th to 75th percentile. The whisker length is set to two inter-quartile ranges.

As a baseline comparison for the predictive power of SPoC components, a linear regression model employing channel-wise bandpower features was evaluated as described in Section 2.6. The resulting distributions of the overall correlation *R*_*all*_ and the performance separability z-AUC are reported in Figure 4. Across all configurations, SPoC delivers a median correlation *R*_*all, med*_ = 0.07 and a separability of z-AUC_*med*_ = 0.54, while on average the regression performs on chance level. While both methods come up with components revealing z-AUC values above chance level, those with the strongest predictive information are generated by the SPoC method.

### 3.3. Single-trial motor performance predictors

**Figure 5:**
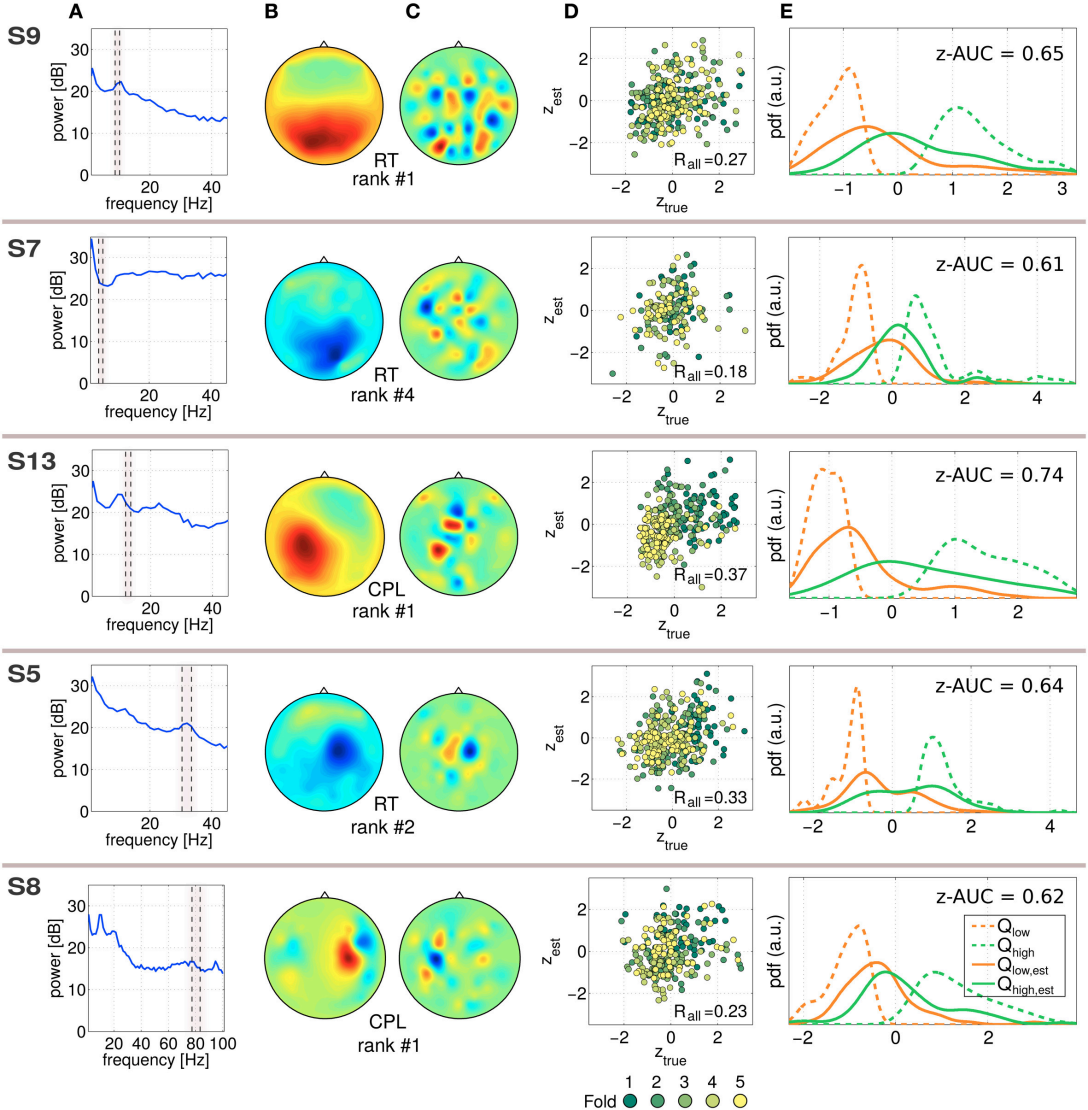
**Characterization of *exemplary* predictive SPoC features**. Each component is characterized line-wise labeled by the used performance metric and the rank according to the full-session filters. **(A)** Power spectrum of the component applied on non-bandpass filtered full data. The frequency band where the component has been trained is marked by the dashed lines. Note that for the component of S8 a broader frequency range is visualized compared to the other examples. **(B)** Spatial activity pattern. **(C)** Filter weights visualized. **(D)** Scatter plot between true labels *z*_*true*_ and the predicted ones *z*_*est*_, color coded by the fold of the chronological cross-validation. **(E)** To illustrate the separability of the prediction, the distribution of *z*_*true*_ values has been split using the corresponding trials of the upper and lower quartiles of *z*_*est*_, which resulted in *Q*_*low, est*_ and *Q*_*high, est*_. As a reference, the extreme quartiles *Q*_*low*_ and *Q*_*high*_ of *z*_*true*_ are also given (dashed curves). In addition, the z-AUC value based on the 50th percentile split is reported.

In Figure 5, five exemplary predictive and robust SPoC components, gained from five different subjects are characterized. Although SPoC components are computed from band-pass filtered data, the resulting filter ***w*** (gained on all available *N*_*e*_ trials) of a component can be re-applied to non-frequency-filtered epoched data. This spectral content of a SPoC component is shown in Figure 5A. The frequency band in which the component was extracted from is indicated by the dashed gray area. Using all available epochs, Figure 5B shows the spatial activity pattern gained via Equation (5). In Figure 5C, the SPoC filter weights on the 2D-scalp projection are shown. The scatter plot in Figure 5D reports on the measured performance metric *z*_*true*_ as a function of the predicted performance *z*_*est*_ according to the CV scheme described in Equation (4). The data points are colored by the fold index (1–5), which corresponds to the temporal order of the session. Fold 1 represents the beginning of the session, fold 5 its end. In addition, the overall correlation *R*_*all*_ reports on the predictive strength of the component. The distributions shown in Figure 5E illustrate the separability of the single-trial performance values *z*_*est*_. For this purpose, the estimated labels *z*_*est*_ have been reduced to the lower and upper quartile. The corresponding true labels *z*_*true*_ were used to compute the quartiles *Q*_*low, est*_ and *Q*_*high, est*_ and were fitted by a kernel distribution (solid lines). In an ideal case, those quartiles would converge toward the extreme quartiles (*Q*_*low*_ and *Q*_*high*_) of *z*_*true*_, which are indicated by dashed lines. As a score of their separability, the score z-AUC as described in Section 2.6 is reported based on the 50th percentile.

The exemplary components in Figure 5 are selected across the investigated frequency range depicted in Figure 2. The predictor of S7 can be assigned to the theta band, those of S9 and S13 correspond to the alpha range, the component for S5 originates from the beta range and the one of S8 was found in the gamma range. Regarding the scatter plots, there are two different types of patterns recognizable: single-trial predictors showing a confined point cloud without a clear trend over time (all examples except for S13), whereas the scatter plot of subject S13 shows a clear trend over the course of the session. The separability plots indicate that the predictive power of a single component nicely matches with the z-AUC value.

### 3.4. Testing the stability of SPoC components

**Figure 6:**
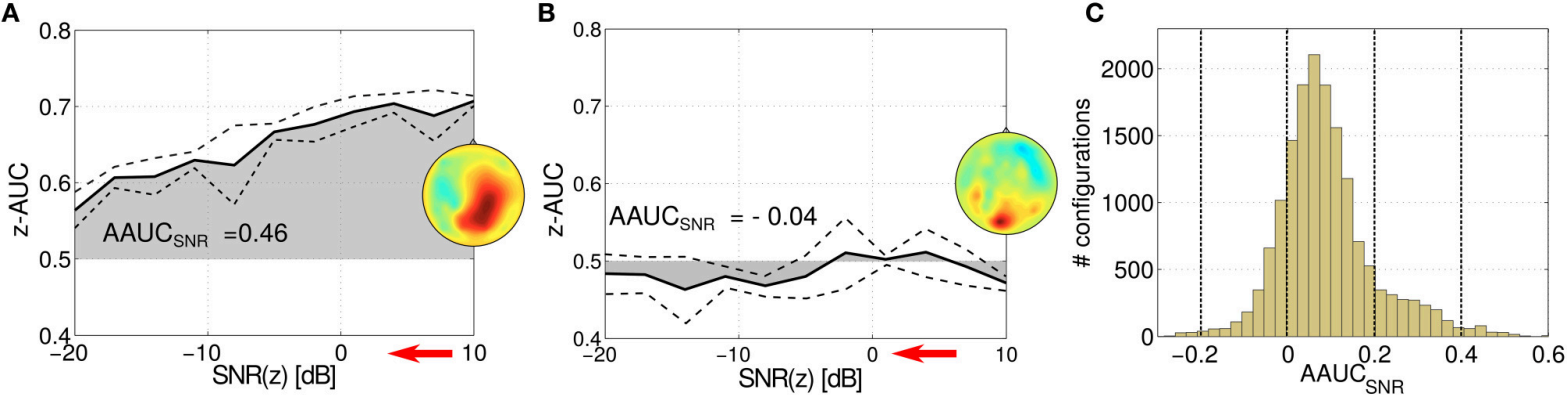
**Stressing the stability of two exemplary SPoC components for two different parameter configurations (A,B)**. While stepwise decreasing the SNR ratio (indicated by the red arrow), z-AUC-values (solid lines) describing the separability of the prediction are plotted together with standard deviations (dashed lines). The area under the z-AUC curve—further on called AAUC_*SNR*_—describes the stability of the component under the challenge of added noise. **(C)** Shows the histogram of all *AAUC*_*SNR*_ scores evaluated for the considered parameter configurations.

The stability of an oscillatory component can be challenged by varying the signal-to-noise ratio (SNR) of the target variable *z*. In Figure 6, the z-AUC score is investigated as a function of the SNR for two parameter configurations. Figure 6A shows a stable component, where z-AUC is expected to decrease, while for a non-informative component in Figure 6B the z-AUC can be expected around the noise floor. Thus, the resulting *area under the* z-AUC *curve* can be assessed as a tool for mapping the stability of the subspace component under challenging noise conditions. In Figure 6C, the distribution of this so-called *AAUC*_*SNR*_ is reported for all evaluated SPoC components across all 18 subjects. The distribution of *AAUC*_*SNR*_ values has its median at 0.07 and is slightly skewed.

### 3.5. Identification of robust and predictive components

**Figure 7:**
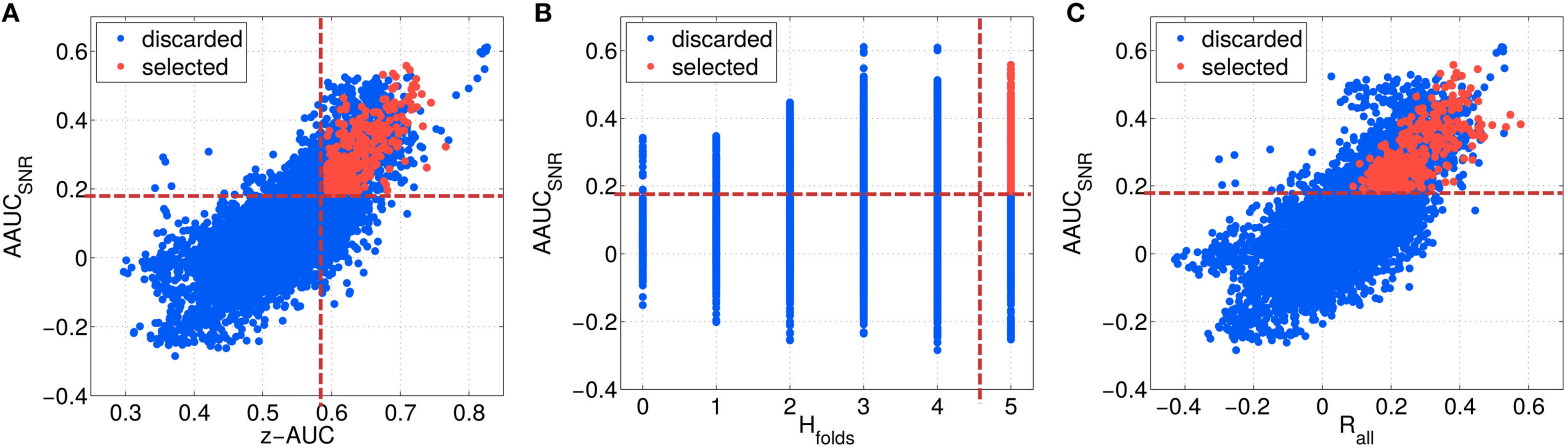
**Characterizing the space of SPoC components by several metrics, which describe their stability and predictive information**. The SNR-challenged AAUC_*SNR*_ is given as a function of the performance separability z-AUC **(A)**, in relation to the homogeneity of the correlation sign *H*_*folds*_ **(B)**, and dependent on the overall correlation *R*_*all*_ **(C)**. Red data points describe the selected SPoC components after applying thresholds (dashed red lines).

As described in Section 2.6, the first highest ranked components of each parameter configuration have been evaluated, resulting in about 12,000 different subspace components. In Figure 7, the configurations are characterized by their stability under noise (AAUC_*SNR*_), which is plotted in Figure 7A as a function of the separability measure z-AUC, in Figure 7B as a function homogeneity of the fold-wise sign of the correlation *H*_*folds*_ and in Figure 7C as a function of the overall correlation *R*_*all*_. A few observations can be made: First, the metrics are not centered at zero. Second, based on all initial configurations (blue data points), AAUC_*SNR*_ correlates with the z-AUC as well as with *R*_*all*_. The largest AAUC_*SNR*_ values are evoked by the most homogeneous fold-wise correlation signs with *H*_*folds*_ ≥ 3.

**Figure 8:**
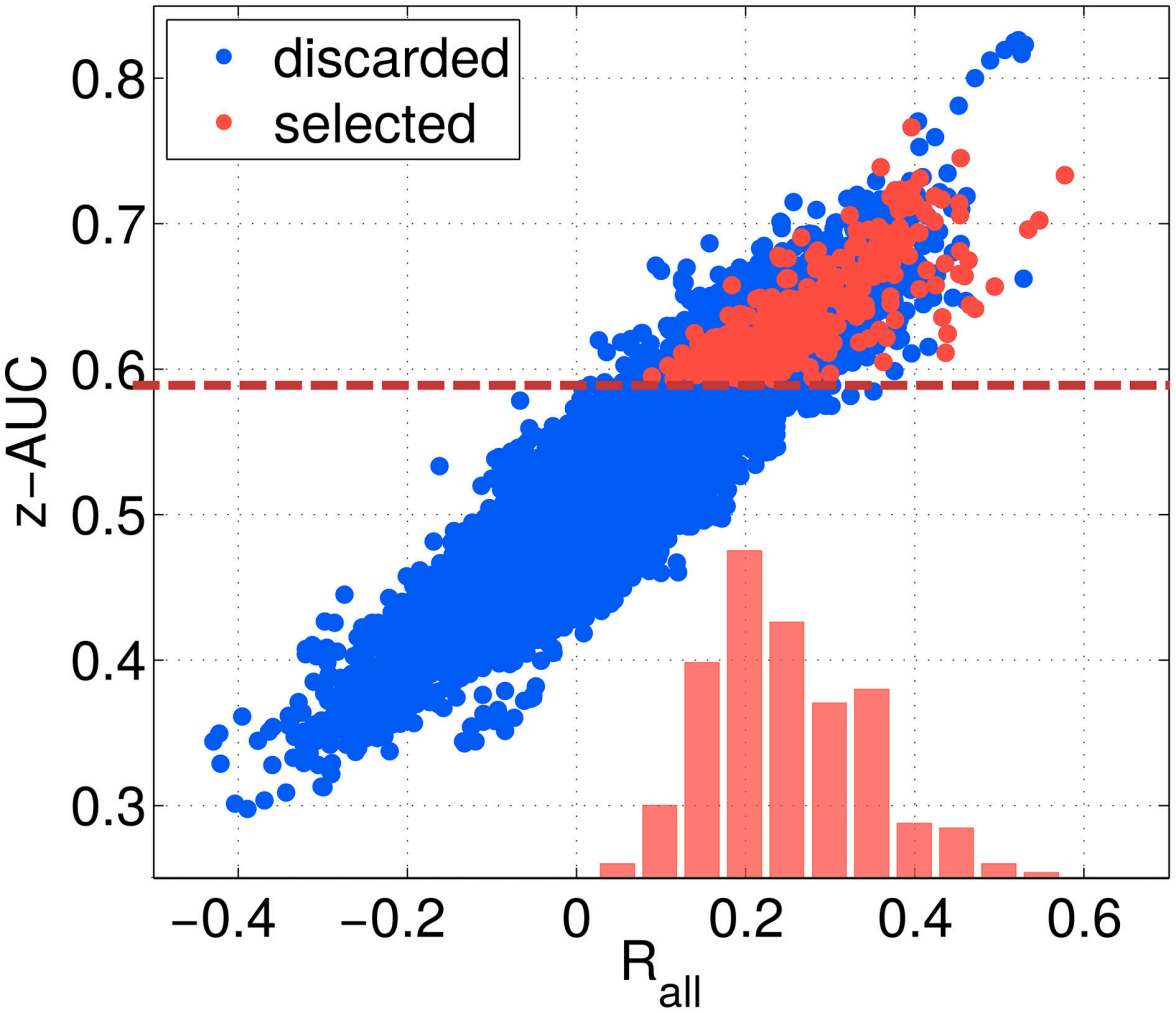
**Relation between separability metric z-AUC and the overall correlation *R*_*all*_ for all SPoC configurations (blue dots) and the selected ones (red dots)**. The dashed red line indicates the threshold z-AUC_*min*_ applied to select most informative components. The red bars indicate the distribution of *R*_*all*_ values for the selected components.

The threshold criteria applied to select the best of the 12,000 subspace components are indicated by red dashed lines, and red dots indicate the components finally selected. As shown in Figure 8, the overall correlation *R*_*all*_ is strongly correlated with the z-AUC metric, such that an additional threshold criterion on *R*_*all*_ was not necessary. The most predictive components achieve a correlation value of up to 0.6, corresponding to ${R_{all}}^{2}=0.36$. Assuming a linear relationship between *z*_*true*_ and *z*_*est*_ as well as normally distributed data, this means that *z*_*est*_ can explain up to 36% of the performance variance contained in *z*_*true*_.

**Figure 9:**
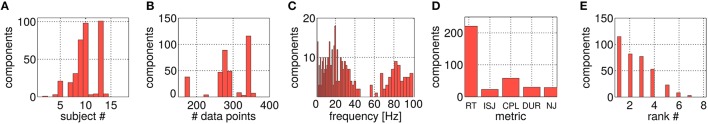
**Histograms of different parameters solely for the selected SPoC components**. **(A)** Shows the assignment over the 18 subjects. **(B)** Gives the allocation over data set sizes *N*_*e*_ (with a lower limit of 150 trials). **(C)** Visualizes the distribution across frequency bands. **(D)** Depicts the spread of components over the five utilized motor performance metrics, while **(E)** shows the split according to their SPoC rank positions.

In Figure 9, all 361 selected components are characterized by histograms in terms of their input parameters. Figure 9A displays the subject-wise grouping. In total, 11 out of 18 subjects contribute at least one component, for three subjects more than 50 configurations survive the selection procedure. Figure 9B shows the histogram over the number of trials available for the offline analysis. Note, that this histogram is dominated by the best three subjects reported in Figure 9A, who contributed a large number of the selected 361 configurations. Figure 9C characterizes the selected components assigned to their underlying frequency band [*f*_0_, *f*_0_ + Δ*f*] (see Figure 2). Most components are gained from the alpha- and beta-band range. Interestingly, robust features detected in the gamma band were dominantly selected for their ability to predict CPL. The slow frequency (<4 Hz) components are dominated by artifactual subspaces. Figure 9D reports on the occurrences of the different performance metrics among the selected components. Most components could be extracted for RT (61%), followed by CPL (16%) while all other metrics contribute almost equally well with 6–8% of the selected components. Figure 9E provides an overview over the SPoC ranks of the surviving components. The rank ordering corresponds to the eigenvalue ordering of the complete data set. As the number of selected components drop with increased rank, the ranking is associated with the information content of the subspace component.

**Figure 10:**
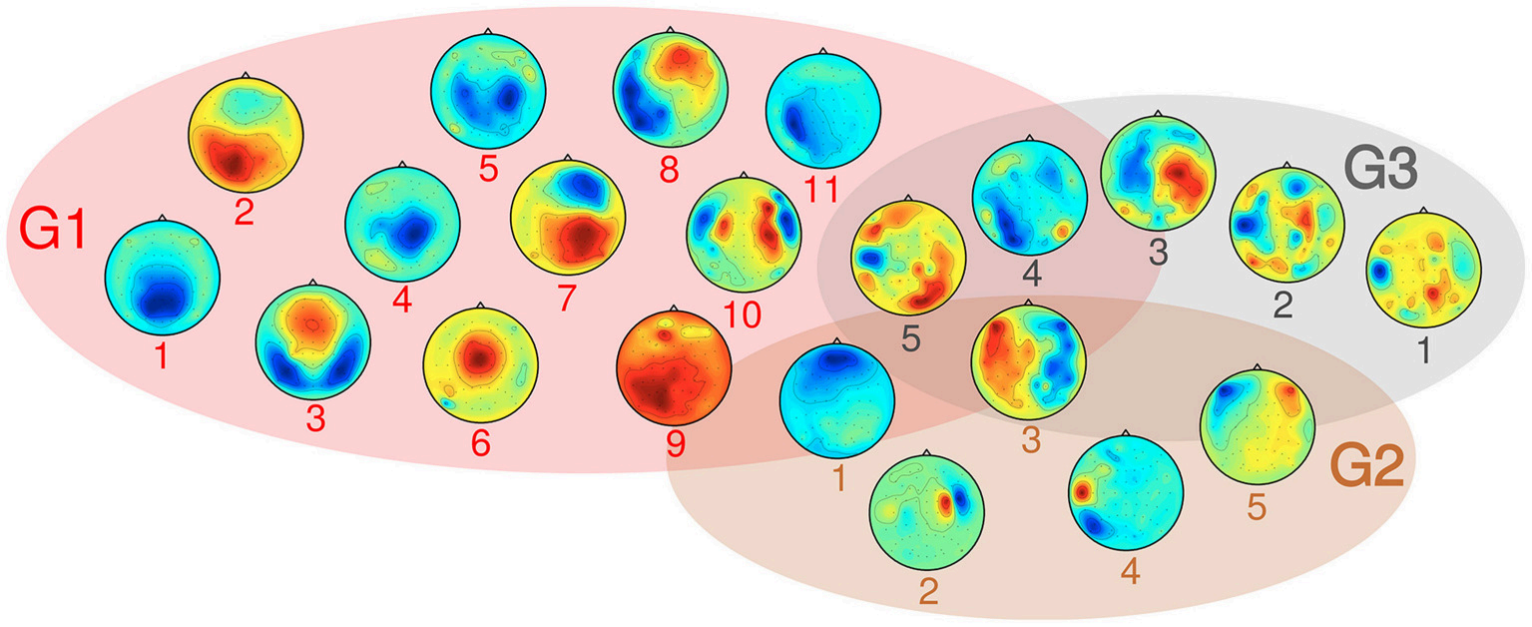
**Overview over typical activity patterns resulting from the selected components, grouped in three categories:** G1 consists of components with neural origin, G2 comprises artifact-related subspaces and G3 captures non-informative components. Details on their parameter configurations are given in Table 1.

SPoC as a linear filtering method allows for a limited neurophysiological interpretation of spatial activity patterns. A representative subset of typical scalp topographies from the selected stable and informative subspaces are plotted in Figure 10. The components were assigned to three groups. About 70% of components fall into group G1, which comprises patterns ranging from activations in occipital, to central or frontal areas. The maximum activity of those components often is found over one of the hemispheres. About 10% of the components fall in group G2. They show patterns of probable non-neural sources and may represent e.g., eye artifacts, muscular activity or single noisy channels. Group G3 comprises noisy topographies. As indicated by patterns in the intersection area of the three groups, mixed components were observed as well. The detailed parameter configurations of each of the plotted components is listed in Table 1.

**Table 1:** **Parameter configurations for components of groups G1, G2 and G3 as visualized in Figure 10**.

	**G1**				**G2**				**G3**			
**Comp. no**.	**Subject**	***f*_0_ [Hz]**	**Metric**	**Rank**	**Subject**	***f*_0_ [Hz]**	**Metric**	**Rank**	**Subject**	***f*_0_ [Hz]**	**Metric**	**Rank**
1	S13	7.9	NJ	2	S14	1.0	RT	2	S12	27.5	ISJ	1
2	S9	8.7	ISJ	3	S11	27.5	ISJ	1	S10	20.3	RT	1
3	S13	7.9	RT	3	S10	5.0	DUR	1	S9	8.7	ISJ	1
4	S13	8.7	NJ	2	S9	39.0	DUR	2	S15	19.2	CPL	1
5	S10	15.3	RT	6	S10	3.7	RT	3	S10	74.4	NJ	2
6	S5	30.4	RT	3								
7	S9	14.4	RT	5								
8	S9	10.2	ISJ	4								
9	S9	18.2	DUR	1								
10	S8	77.4	CPL	4								
11	S13	74.4	CPL	1								

## 4. Discussion

We hypothesized that subject-specific pre-trial brain signals contain information which allows to partially explain and temporally predict the trial-by-trial variability of the upcoming motor performance in SVIPT. To test the hypothesis, we developed a workflow which is capable to extract informative oscillatory EEG subspace components and to identify the most robust ones. Simulating an online application, our analysis revealed strong evidence that the band power of the selected components is predictive for the single-trial SVIPT performance. Major findings were that these components indeed exist, but need to be optimized for individual users. With 11 out of 18, not all, but a majority of the subjects revealed the desired informative features.

In the following we will first discuss the decision to utilize SPoC instead of other alternative analysis methods. In this context, the proposed selection procedure and the stability of SPoC components over time is discussed, with a special focus onto the role of SNR, frequency and the illiteracy phenomenon. In addition, the detected components will be related to existing literature and characterized on a group-level with respect to the covered frequency bands, sub-processes reflected by the components and the time courses revealed. Before concluding, we will describe a neuroergonomically enhanced rehabilitation paradigm as a possible use case of our contribution.

### 4.1. SPoC and its alternatives

Designing the data analysis workflow, we built upon our background in BCI. Accordingly, we carefully selected algorithmic building blocks only, if they can be applied in single-trial analysis [e.g., the application of the spatial SPoC filter according to Equation (4)]. This decision should simplify the translation of the presented workflow to closed-loop experiments in the future. The choice of the supervised SPoC algorithm for extracting informative components is supported by its good performance compared to a supervised linear regression of bandpower features on the sensor level (see Section 3.2). This is in accordance with findings of Dähne et al. (2014). On data from an auditory steady-state evoked potential paradigm, these authors reported better results for SPoC compared to both, linear regression and an unsupervised subspace decomposition using independent component analysis (ICA). SPoC does not reconstruct sources of the brain, but instead performs a supervised subspace decomposition. Thus, a SPoC subspace component can not be expected to correspond to a single physical source or even a dipole source (even though such SPoC components are possible). Theoretically even several spread-out brain areas may contribute to a single SPoC component, if they share oscillatory activity which co-varies over time with the labels. The choice between SPoC and source reconstruction approaches (Gonzalez Andino et al., 2005) represents a trade-off—while the latter may facilitate the interpretation of results, SPoC components avoid several of the drawbacks mentioned in Section 2.5. As our workflow was aligned in terms of applicability for single-trial online paradigms, our decision was biased toward SPoC.

### 4.2. Selection criteria for robust and predictive components

Over-fitting is a general issue for supervised methods and for SPoC in particular, as no form of regularization was applied. This requires some form of *post-hoc* selection of SPoC components. The situation is aggravated, as SPoC returns full rank filter matrices, which result in a very large numbers of subspaces. However, only a fraction of these can be expected to be informative about the labels. As robustness over time as well as with respect to label noise are important criteria for the potential closed-loop applicability of a component, a single selection criterion (e.g., a threshold on the correlation value) is not sufficient. By that, we selected three criteria (see Section 2.6), which suited best these requirements. Out of the initial five selection criteria, the two scores *R*_*all*_ and *R*_*folds*_ turned out to be beneficial for characterizing the extracted components. Thus, they were omitted for the selection process, since a strong correlation between z-AUC and *R*_*all*_ was observed (see Figure 8). The same holds for the correlation between z-AUC and *R*_*folds*_ (not shown).

An alternative to the current selection procedure would be to relax the thresholds and combine it with additional methods to judge the plausibility of the remaining components *post-hoc*. For ICA components, workflows have been proposed, such as MARA, an automatic classification of artifactual components by Winkler et al. (2014). MARA uses features based on topology, time-frequency analysis and source reconstruction. Similar approaches have been proposed by Daly et al. (2015) and Grosse-Wentrup et al. (2013).

### 4.3. Influence of SNR on SPoC components

By applying rather strict selection criteria, weaker but still informative components may have been removed. As a result, the data of some subjects did not reveal informative pre-go oscillatory components. Reasons may be a lower SNR of their data, which hides potential informative content from the SPoC analysis, especially in combination with the limited number of trials used. The work of Castaño-Candamil et al. (2015a) on robustness testing of SPoC components backs this interpretation. In this case, future improvements may be expected by regularization techniques introduced to SPoC, a reduction of the dimensionality prior to applying SPoC, using more data or from transfer learning approaches. However, we can not exclude that informative oscillatory components may not be visible to the EEG or may be absent in some subjects. This problem has been described as BCI “illiteracy.” It has predominantly been studied in the context of motor imagery paradigms for the control of BCI applications (Hammer et al., 2012), where decoding the imagery class usually is not possible for a subset of subjects. The BCI illiteracy problem was tackled by novel experimental setups like hybrid BCI paradigms (Allison et al., 2012; Müller-Putz et al., 2015), but could also be alleviated by more advanced decoding methods (Sannelli et al., 2010).

### 4.4. Rank stability of SPoC components over time

**Figure 11:**
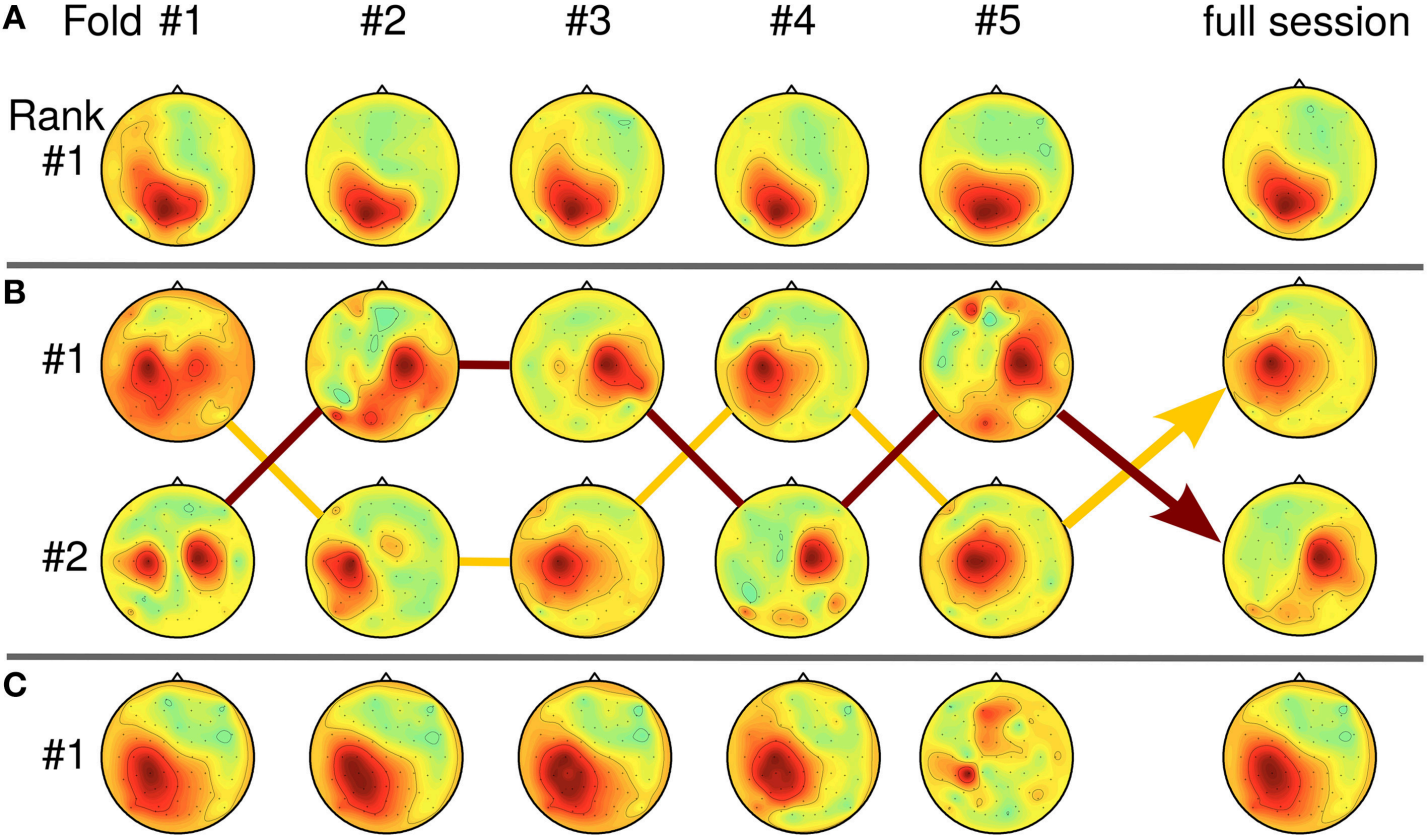
**Relation between SPoC rank stability and pattern homogeneity over five cross-validation folds (chronological order)**. **(A)** Stationary case: component is first-ranked across all five folds (data of subject S9, *f* = [9.4, 11] Hz, RT). **(B)** Rank switching: Two almost stable components switch rank positions between folds (S5, *f* = [27.5, 30.3] Hz, RT). Lines connect the corresponding topologies. **(C)** Intensity variation: intensity of first-ranked component decreases over time folds (S13, *f* = [13.6, 15.3] Hz, CPL).

In Section 4.3, the relation between SPoC solutions and the SNR of the data has been touched. As SPoC ranks the detected components according to their covariance values, solutions may seem unstable when only the first-ranked component is considered. In real-life data sets, variation of the SNR over time can induce rank switches or mixed components. Tracking a component over multiple runs of the subspace decomposition method is a challenging task, especially as mixtures theoretically can not be distinguished from a single source. However, as similar problems arise for online learning of blind source separation methods like ICA, practical solutions are available (Hsu et al., 2015). Figure 11 gives examples of stable, stationary components (Figure 11A) and of unstable SPoC components (Figures 11B,C), both observed over the five chronological cross-validation folds. Instable components may be evoked if the stationarity assumption of SPoC is violated e.g., by slow temporal intensity variations due to user learning. For Figure 11B, arrows indicate a possible path through rank positions across folds by connecting corresponding components. Please observe, that SPoC generates cases with even severe variation between folds as those depicted in Figures 11B,C. However, such components typically have been removed during the selection process. While mixed, yet stable components may be hard to interpret, they can still be useful for predicting the task performance.

**Figure 12:**
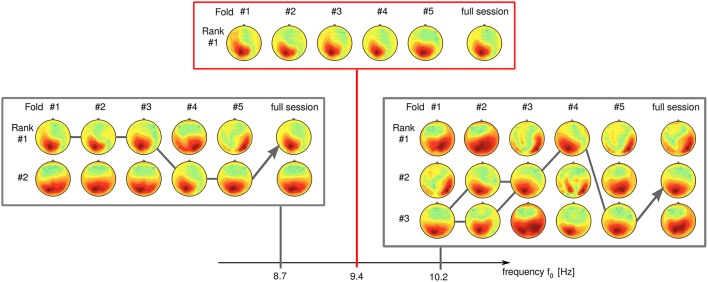
**Influence of the frequency band upon the rank stability**. While stable at *f*_0_ = 9.4 Hz, a component develops rank instability with slight increase/decrease of its frequency band (data of subject S9).

We have observed a high sensitivity of SPoC for small differences in the frequency parameters. Seemingly unstable components which display rank switching behavior (see Figure 12 at 8.7 Hz) can sometimes be stabilized by slightly changing the frequency, e.g., to 9.4 Hz in this example. Further increase of the frequency to 10.2 Hz again induces instability in this example.

### 4.5. Characterization of informative SPoC components and sub-processes

The proposed SPoC workflow delivers a diverse set of oscillatory components, which vary in their topological patterns as well as in their underlying frequency band. This is not surprising, since SVIPT requires the interaction of several cognitive sub-processes in order to reach a good overall performance. For each sub-process, one or more specific neural features may exist, with all of them being informative about the overall outcome of the complex task.

The best components differ between subjects and predominantly occur in the alpha band, followed by beta and gamma band. Our findings are supported by informative features in the alpha and beta-range observed during pre-movement intervals of a hand grasping task (Zaepffel et al., 2013; Meyer et al., 2014; Yang et al., 2014). Furthermore, the informative frequency ranges for SVIPT are comparable to those reported for attention related tasks (Gonzalez Andino et al., 2005; Hoogenboom et al., 2010; van Ede et al., 2012). We obtained best results when using RT as a performance metric, which supports our earlier findings on disjunct data from younger subjects (Castaño-Candamil et al., 2015c; Meinel et al., 2015). RT of course does not automatically lead to a successful trial, but it can be seen as an indicator for a quick ramp-up phase and alertness. For fewer users, presumably those with highest SNR characteristics, informative oscillatory features could be identified for other performance metrics of the force task, too.

Comparing the topological plots of group G1 in Figure 10 with those reported in literature, it can be observed that many of them resemble patterns emerging for motor imagery tasks in BCI (Krauledat et al., 2008). These often display a clear maximum of activity in channels located over one of the sensorimotor areas (cp. pattern 5 of G1 in Figure 10 and the pattern of S5 in Figure 5) or are located centrally over both hemispheres. While similarity of patterns are by no way a proof for an origin of these oscillatory components in the sensorimotor cortices, the hand force action required to succeed in the SVIPT task would allow for such components.

Other components show a maximum intensity over parietal and occipital areas and may reflect the involvement of the visual system in the SVIPT task. Pattern 2 of Figure 10 and patterns in Figure 11A display a lateralization similar to patterns reported for *directed* and covert visual attention processes (Hanslmayr et al., 2007; Horschig et al., 2014a). Components with a centrally located maximum (cp. pattern 1 of Figure 10 or the pattern of S9 in Figure 5), or with double wing shapes (e.g., pattern 3 of Figure 10) resemble components reported for generalized visual attention processes (van Dijk et al., 2008; Meyer et al., 2014). Again, most of these rather clear patterns originate from the alpha frequency band.

While the relevance of several of the selected components cannot be fully interpreted, we do consider these features as added value for neurologists, e.g., by tracking the power time course over sessions for a subject-specific component. Further insight into underlying sub-processes and participating brain areas may be obtained from a *post-hoc* source reconstruction applied upon single SPoC subspaces.

### 4.6. Behavioral variability on different time scales

**Figure 13:**
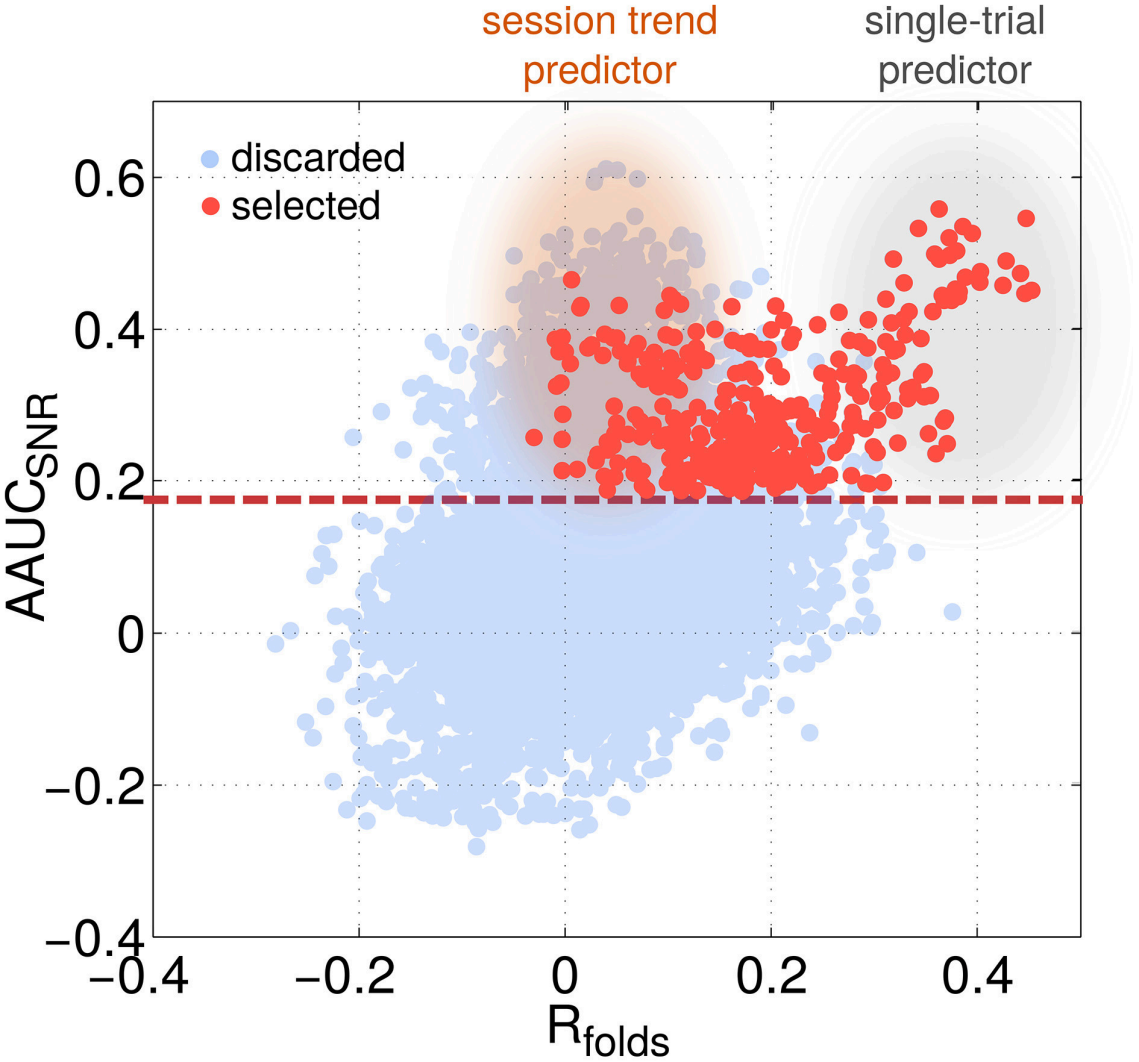
**The identification of session trends vs. single-trial variations of performance is possible by localizing a predictor's characteristic with respect to two selection criteria**. The scatter plot visualizes AAUC_SNR_ as a function of the mean correlation value across folds *R*_*folds*_ for all configurations (light blue) and the selected ones only (red). Two classes of predictors can be identified: single-trial predictors showing a high *R*_*folds*_ value while session-trend predictors show a very low *R*_*folds*_ value.

Independent of the choice of the exact motor task, subjects generally display two types of performance variations (Chaisanguanthum et al., 2014). First, a large trial-to-trial performance variability is observed from behavioral data. Second, slow performance drifts can occur over the course of a session. Accordingly, SPoC can deliver components, which reflect either one of the two types of performance variations. To tell them apart, a comparison between *R*_*all*_ and *R*_*folds*_ is helpful. High values for *R*_*all*_, but low for *R*_*folds*_ indicate a session trend. If both are high, then the component is informative for trial-by-trial variation (see *single-trial predictors* and *session trend predictors* in Figure 13 as well as the examples given in Figure 5).

For the purpose of brain-state-informed closed-loop experimenting, single-trial predictors may be more suitable. Session trend predictors, however, may still be useful for pre-cleaning the performance labels. While session trend predictors may reflect an increasing fatigue or a learning effect, it is much harder to determine underlying mechanisms, which cause the rapidly changing trial-to-trial performance of the single-trial predictors (Wu et al., 2014; Hadjiosif and Smith, 2015; Osu et al., 2015). However, our identified components reveal strong evidence that the pre-trial brain activity is partially informative about trail-by-trial variability of motor performance. This in accordance with Churchland et al. (2006) who reported on monkey experiments that at least 30% of behavioral variability could be explained by the fluctuations of preparatory neural activity in the dorsal premotor cortex. However, Chaisanguanthum et al. (2014) stated only a weak relationship between motor cortex activity (PMd/M1) in monkeys and trial-wise fluctuations of behavior.

### 4.7. Closed-loop experimenting as neuroergonomical application

The predictive EEG features are extracted from a pre-go interval of each trial. Our pipeline carefully simulated an online scenario, but this approximation of course can not replace the evaluation within a future online study. However, the informative trial-by-trial performance predictors may serve to enhance the neureorgonomical needs of motor rehabilitation scenarios. Since motor performance variability was reported to become larger for stroke patients (Lodha et al., 2010), applying identified patient-specific components within brain-state dependent closed-loop experimenting may enable to causally influence their performance e.g., by manipulating difficulty levels in motor rehabilitation paradigms. So far, BCI methods in stroke rehabilitation (Ang and Guan, 2013) have been used to detect the attempted movement of the affected hand by analyzing informative ERD/ERS features of the EEG and subsequently close the feedback loop for the patient either by triggering a simulated hand movement on a screen (Pichiorri et al., 2015) or by triggering a passive movement of the affected hand, e.g., via an external robotic device or an active orthesis (Ramos-Murguialday et al., 2013).

When implemented in future closed-loop applications, it may be worth to combine SPoC features across multiple frequency bands e.g., by a regression approach. This might allow for enhancing the trial-wise performance prediction, in case the information contained in different frequency bands is independent. Similarly, the combination of predictors based on different performance metrics might serve to gain an enhanced performance estimate.

## 5. Conclusion

In summary, we have shown that the proposed workflow is a suitable basis to identify subject-specific single-trial based neural markers which are predictive for the performance of an upcoming motor task. Those predictors may be valuable building blocks for neuroergonomic applications since they are informative about the status of the visual subsystem as well as the sub-processes involved in hand motor control. Moreover, exploiting those features in future closed-loop experimenting, e.g., by temporal gating of upcoming trials, they will allow for brain-state-informed rehabilitation paradigms. Furthermore, the group-level analysis motivated to utilize our workflow to gain a better understanding of trial-to-trial variations of cognitive sub-processes, which are relevant for a successful rehabilitation outcome.

## Author contributions

Conceived and designed the experiments: AM, SC, JR, and MT. Performed the experiments: AM, SC, and MT. Analyzed the data: AM and MT. Contributed reagents/materials/analysis tools: AM, SC, JR, and MT. Wrote the paper: AM and MT.

### Conflict of interest statement

The authors declare that the research was conducted in the absence of any commercial or financial relationships that could be construed as a potential conflict of interest.

## Footnotes

^1^The components are sorted according to their eigenvalues. In case of SPoC_λ_, they equal to the covariance between the bandpower features and the target variable.
